# Epigallocatechin Gallate in *Camellia sinensis* Ameliorates Skin Aging by Reducing Mitochondrial ROS Production

**DOI:** 10.3390/ph18050612

**Published:** 2025-04-23

**Authors:** Ji Ho Park, Eun Young Jeong, Ye Hyang Kim, So Yoon Cha, Ha Yeon Kim, Yeon Kyung Nam, Jin Seong Park, So Yeon Kim, Yoo Jin Lee, Jee Hee Yoon, Byeonghyeon So, Duyeol Kim, Minseon Kim, Youngjoo Byun, Yun Haeng Lee, Song Seok Shin, Joon Tae Park

**Affiliations:** 1 Division of Life Sciences, College of Life Sciences and Bioengineering, Incheon National University, Incheon 22012, Republic of Korea; 202428002@inu.ac.kr (J.H.P.); juli9709@inu.ac.kr (Y.J.L.); yoojn0905@inu.ac.kr (J.H.Y.); tundra@inu.ac.kr (B.S.); papaya1130@inu.ac.kr (D.K.); alstjs0323@inu.ac.kr (M.K.); 2 Hyundai Bioland Co., Ltd., 22, Osongsaengmyeong 2-ro, Osong-eup, Heungdeok-gu, Cheongju-si 28162, Republic of Korea; eyjeong99@hyundaibioland.co.kr (E.Y.J.); dpgid27@hyundaibioland.co.kr (Y.H.K.); sycha@hyundaibioland.co.kr (S.Y.C.); hayeonkim@hyundaibioland.co.kr (H.Y.K.); namyk@hyundaibioland.co.kr (Y.K.N.); wlstjd1324@hyundaibioland.co.kr (J.S.P.); sysy@hyundaibioland.co.kr (S.Y.K.); 3 College of Pharmacy, Korea University, Sejong 30019, Republic of Korea; yjbyun1@korea.ac.kr; 4 Interdisciplinary Major Program in Innovative Pharmaceutical Sciences, Korea University, Sejong 30019, Republic of Korea; 5 Convergence Research Center for Insect Vectors, Incheon National University, Incheon 22012, Republic of Korea

**Keywords:** *Camellia sinensis*, reactive oxygen species (ROS), senescence rejuvenation, skin aging recovery

## Abstract

**Background:** Reactive oxygen species (ROS) generated by mitochondrial dysfunction damage cellular organelles and contribute to skin aging. Therefore, strategies to reduce mitochondrial ROS production are considered important for alleviating skin aging, but no effective methods have been identified. **Methods:** In this study, we evaluated substances utilized as cosmetic ingredients and discovered *Camellia sinensis* (*C. sinensis*) as a substance that reduces mitochondrial ROS levels. **Results:** *C. sinensis* extracts were found to act as senolytics that selectively kill senescent fibroblasts containing dysfunctional mitochondria. In addition, *C. sinensis* extracts facilitated efficient electron transport in the mitochondrial electron transport chain (ETC) by increasing the efficiency of oxidative phosphorylation (OXPHOS), thereby reducing mitochondrial ROS production, a byproduct of the inefficient ETC. This novel mechanism of *C. sinensis* extracts led to the restoration of skin aging and the skin barrier. Furthermore, epigallocatechin gallate (EGCG) was identified as an active ingredient that plays a key role in *C. sinensis* extract-mediated skin aging recovery. Indeed, similar to *C. sinensis* extracts, EGCG reduced ROS and improved skin aging in an artificial skin model. **Conclusions:** Our data uncovered a novel mechanism by which *C. sinensis* extract reverses skin aging by reducing mitochondrial ROS production via selective senescent cell death/increased OXPHOS efficiency. Our results suggest that *C. sinensis* extract or EGCG may be used as a therapeutic agent to reverse skin aging in clinical and cosmetic applications.

## 1. Introduction

Human skin is composed of the epidermis, dermis, smooth muscle, and adipose tissue and functions as a barrier to protect against pathogens, while maintaining body temperature [1]. Keratinocytes are the main cells found in the skin epidermal layer [2]. Keratinocyte synthesis decreases with age, increasing the sensitivity to ultraviolet (UV) radiation [3]. Fibroblasts, the main cells of the dermis, also change with age, producing less collagen, which reduces the skin barrier function [4]. These alterations in the epidermis and dermis ultimately act as the main cause of skin aging [4]. Skin aging is characterized by functional alterations in organelles, particularly mitochondria [5]. As skin ages, mitochondria undergo structural changes, leading to the accumulation of defective mitochondria, which increase significantly in size and volume [6]. Defective mitochondria leak electrons from the electron transport chain (ETC), generating ROS, which contain radicals such as superoxide anion (O_2_^●–^) and hydroxyl radical (^●^OH) [7]. In addition to the main source of ROS generation, defective mitochondria are susceptible to ROS-mediated oxidative stress, which amplifies mitochondrial ROS production. Increased oxidative stress leads to lipid peroxidation, protein oxidation, and collagen/elastin chain cleavage, which in turn weakens the skin barrier and worsens skin aging [6]. Consequently, strategies to reduce mitochondrial ROS production have been widely attempted as a beneficial treatment for skin aging [8,9].

Inefficient electron transport in the mitochondrial ETC is one of the major causes of ROS production [10]. When electrons leak from ETC components, oxygen is converted to O_2_^●−^ [10]. Since the mitochondrial ETC drives oxidative phosphorylation (OXPHOS) by transferring electrons and protons from the mitochondrial matrix to the mitochondrial intermembrane space [11], the efficiency of electron transport in ETC can be indirectly quantified using OXPHOS efficiency [12].

Skin is the epithelial tissue where the signs of aging are most noticeable [13]. Skin aging is characterized by a decrease in collagen protein synthesis, which leads to a loss of structural support and a decline in skin barrier function [14]. Collagens type I and III are necessary for skin tissue regeneration, with collagen type III synthesis increasing in the early stages of skin regeneration and collagen type I synthesis increasing in the late stages [15]. Collagen type IV is an important component of the basement membrane that provides structural support to skin tissue and helps skin barrier function [16].

Senescent cells secrete inflammatory and matrix-degrading factors that alter tissue function and structure [17]. Senotherapy can be divided into senomorphics and senolytics [18]. Senomorphics increase cell proliferation while maintaining the young cell pool without inducing apoptosis of senescent cells [18]. However, senolytics selectively remove senescent cells that emit inflammatory signals that can disrupt normal tissue function [19]. Senolytics kill senescent cells by inducing apoptosis while sparing non-senescent cells [19]. Several senolytics have been widely used to treat aging. Specifically, quercetin is a plant flavonoid with senolytic properties [20]. Quercetin reduced the number of senescence-associated β-galactosidase (SA-β-gal) positive cells, a characteristic of senescent cells, by suppressing inflammatory cytokines [21]. Fisetin, a flavonoid with powerful antioxidant properties, has also been shown to have senolytic effects, removing senescent cells without harming healthy cells [22]. Fisetin has shown promise as an anti-aging treatment by extending the lifespan of yeast, fruit flies, and mice by more than 50%, 20%, and 10%, respectively [22]. These potentials have made senolytics a cutting-edge field in aging research and regenerative medicine, but research into the underlying mechanisms is needed.

*Camellia sinensis* (*C. sinensis*), a plant belonging to the *Theaceae* family, has been a traditionally used medicinal plant due to its anti-viral and antimicrobial properties [23]. For example, *C. sinensis* has been shown to have antiviral effects against HIV by inhibiting the interaction of the HIV envelope with host cell receptors [24]. *C. sinensis* has also shown excellent antibacterial activity against a wide range of Gram-positive/negative bacteria [25]. In addition to its antiviral and antibacterial properties, *C. sinensis* has been used as a cosmetic ingredient. In particular, *C. sinensis* has been shown to be beneficial in terms of skin soothing and damage repair [26]. However, the fundamental mechanisms and active ingredients of *C. sinensis* extract have not yet been explored. Understanding the mechanisms of the anti-aging effects of *C. sinensis* and identifying its active ingredients may help in its more effective use as a cosmetic additive and skin care product.

Mitochondria are the major organelles that generate free radicals that damage cells [10]. More than 90% of oxygen is used in mitochondria, and 1–5% is converted to ROS by the ETC complex [10]. The most commonly used reagents to detect ROS are 2′,7′-dichlorofluorescein diacetate or 2,7-dichlorofluorescein acetoxymethyl ester [27,28,29,30]. However, since both reagents are oxidized by hydrogen peroxide, a type of ROS present in the cytoplasm, both reagents can detect hydrogen peroxide but cannot specifically detect ROS generated in mitochondria [29,31]. Recently, dihydrorhodamine 123 (DHR123) has been developed to detect mitochondrial ROS. Rhodamine 123, a dye that selectively stains mitochondria and passively diffuses across the cell membrane, is reduced to form DHR123 [32]. DHR123 is then oxidized to cationic rhodamine 123 upon binding to mitochondrial ROS, allowing the detection of mitochondrial ROS levels [33].

In this study, we identified that *C. sinensis* extract acts as an antioxidant that significantly reduces mitochondrial ROS production in senescent fibroblasts. The decrease in mitochondrial ROS production by *C. sinensis* extract was achieved by the selective apoptosis of senescent fibroblasts and subsequent increase in OXPHOS efficiency. The novel mechanism induced by *C. sinensis* extract was found to play an important role in the recovery of skin aging. Furthermore, we found which active components of *C. sinensis* extract are most important in exhibiting these effects. Here, we propose a unique mechanism of *C. sinensis* extract and its active components for the recovery of skin aging.

## 2. Results

### 2.1. Effect of C. sinensis Extract on Reducing Mitochondrial ROS Levels

**Figure 1:**
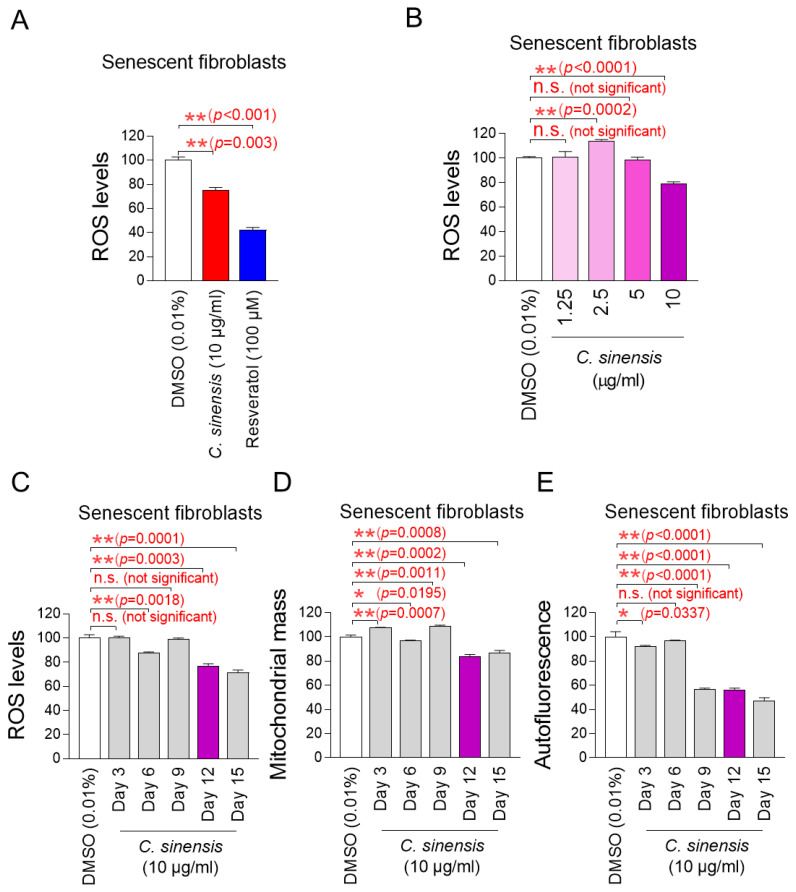
Effect of *C. sinensis* extract on reducing mitochondrial ROS levels. (**A**) Senescent fibroblasts were treated with dimethyl sulfoxide (DMSO) (0.01%), *Camellia sinensis* (*C. sinensis*) extract (10 μg/mL), or resveratrol (100 μM) for 12 days. Use of dihydrorhodamine 123 (DHR123) for fluorescence-activated cell sorting (FACS) analysis. ** *p* < 0.01, Student’s *t*-test. Mean ± S.D., *n* = 3. (**B**) Senescent fibroblasts were treated with DMSO (0.01%) or different concentrations of *C. sinensis* extract (1.25, 2.5, 5, and 10 µg/mL) for 12 days. Use of dihydrorhodamine 123 (DHR123) for FACS analysis. n.s. (not significant), ** *p* < 0.01, Student’s *t*-test. Mean ± S.D., *n* = 3. (**C**) Senescent fibroblasts were treated with DMSO (0.01%) or *Camellia sinensis* (*C. sinensis*) extract (10 μg/mL) for 3, 6, 9, 12, and 15 days. Use of dihydrorhodamine 123 (DHR123) for FACS analysis. n.s. (not significant), ** *p* < 0.01, Student’s *t*-test. Mean ± S.D., *n* = 3. (**D**) Senescent fibroblasts were treated with DMSO (0.01%) or *Camellia sinensis* (*C. sinensis*) extract (10 μg/mL) for 3, 6, 9, 12, and 15 days. Use of MitoTracker™ Deep Red FM Dye for FACS analysis. n.s. (not significant), * *p* < 0.05, ** *p* < 0.01, Student’s *t*-test. Mean ± S.D., *n* = 3. (**E**) Senescent fibroblasts were treated with DMSO (0.01%) or *Camellia sinensis* (*C. sinensis*) extract (10 μg/mL) for 3, 6, 9, 12, and 15 days. Autofluorescence was measured using FACS. n.s. (not significant), * *p* < 0.05, ** *p* < 0.01, Student’s *t*-test. Mean ± S.D., *n* = 3.

We explored potential substances that can effectively reduce mitochondrial ROS levels in senescent fibroblasts using *C. sinensis* extract. Senescent fibroblasts were treated with *C. sinensis* extract (10 µg/mL) for 12 days. Then, DHR123, which can accurately detect ROS in mitochondria, was used to evaluate how each extract affected ROS levels [32]. Resveratrol, a potent antioxidant, was employed as a positive control [34]. As expected, resveratrol significantly reduced mitochondrial ROS levels compared to the DMSO control. *C. sinensis* extract significantly reduced ROS levels compared to the DMSO control (Figure 1A). These results showed that *C. sinensis* extract, which is known to be good for skin health, also exhibited antioxidant activity.

The finding that *C. sinensis* extract was beneficial in reducing ROS prompted us to conduct experiments to establish the optimal concentration of *C. sinensis* extract for reducing ROS. To determine the optimal concentration, senescent fibroblasts were with *C. sinensis* extract at concentrations of 1.25, 2.5, 5, and 10 μg/mL. At concentrations of 1.25 and 5 μg/mL, *C. sinensis* extract did not significantly affect ROS levels compared to the DMSO control (Figure 1B). In contrast, at a concentration of 2.5 μg/mL, *C. sinensis* extract significantly increased ROS levels compared to the DMSO control (Figure 1B). However, the significant reduction in ROS effect was observed when senescent fibroblasts were treated with *C. sinensis* extract only at the concentration of 10 μg/mL (Figure 1B). This result may be because the concentrations of *C. sinensis* extract (1.25, 2.5, and 5 μg/mL) did not approach the availability of intracellular receptors, which did not lead to the desired ROS reduction effect [35]. Thus, the optimal concentration of *C. sinensis* extract was determined to be 10 μg/mL.

The discovery of the optimal concentration of *C. sinensis* extract led to a study on how long *C. sinensis* extract should be administered to senescent fibroblasts to achieve the desired effect. In addition to ROS, mitochondrial mass and lipofuscin, known as senescence-associated phenotypes, were measured at 3, 6, 9, 12, and 15 days after *C. sinensis* extract administration. Treatment with *C. sinensis* extract for 3 days did not reduce ROS in senescent fibroblasts compared to the DMSO control (Figure 1C). However, treatment with *C. sinensis* extract for 6 days significantly reduced ROS compared to the DMSO control (Figure 1C). On the contrary, treatment with *C. sinensis* extract for 9 days did not reduce ROS compared to the DMSO control (Figure 1C). However, treatment with *C. sinensis* extract for 12 and 15 days significantly reduced ROS levels compared to the DMSO control (Figure 1C).

Then, the mitochondrial mass was measured, because one of the main characteristics of senescence is an increase in mitochondrial mass [7]. Treatment with *C. sinensis* extract for 3 days did not reduce mitochondrial mass in senescent fibroblasts compared to the DMSO control (Figure 1D). However, treatment with *C. sinensis* extract for 6 days significantly reduced mitochondrial mass compared to the DMSO control (Figure 1D). Conversely, after 9 days of *C. sinensis* extract treatment, the mitochondrial mass significantly increased compared to the DMSO control (Figure 1D). However, treatment with *C. sinensis* extract for 12 and 15 days significantly reduced mitochondrial mass compared to the DMSO control (Figure 1D).

Finally, the optimal time of *C. sinensis* extract treatment was determined by measuring lipofuscin. Lipofuscin, one of the main characteristics of senescence, was measured by measuring autofluorescence [36]. Treatment with *C. sinensis* extract for 3 days significantly reduced autofluorescence in senescent fibroblasts compared to the DMSO control (Figure 1E). However, treatment with *C. sinensis* extract for 6 days did not reduce autofluorescence compared to the DMSO control (Figure 1E). Treatment with *C. sinensis* extract for 9, 12, and 15 days significantly reduced autofluorescence compared to the DMSO control (Figure 1E).

In summary, treatment with *C. sinensis* extract for 12 and 15 days significantly reduced ROS levels, mitochondrial mass, and lipofuscin compared to the DMSO control. Therefore, 12 days, which is the shorter treatment period among 12 and 15 days, was determined to be the optimal time.

### 2.2. C. sinensis Extract Selectively Kills Senescent Fibroblasts by Inducing Apoptosis

**Figure 2:**
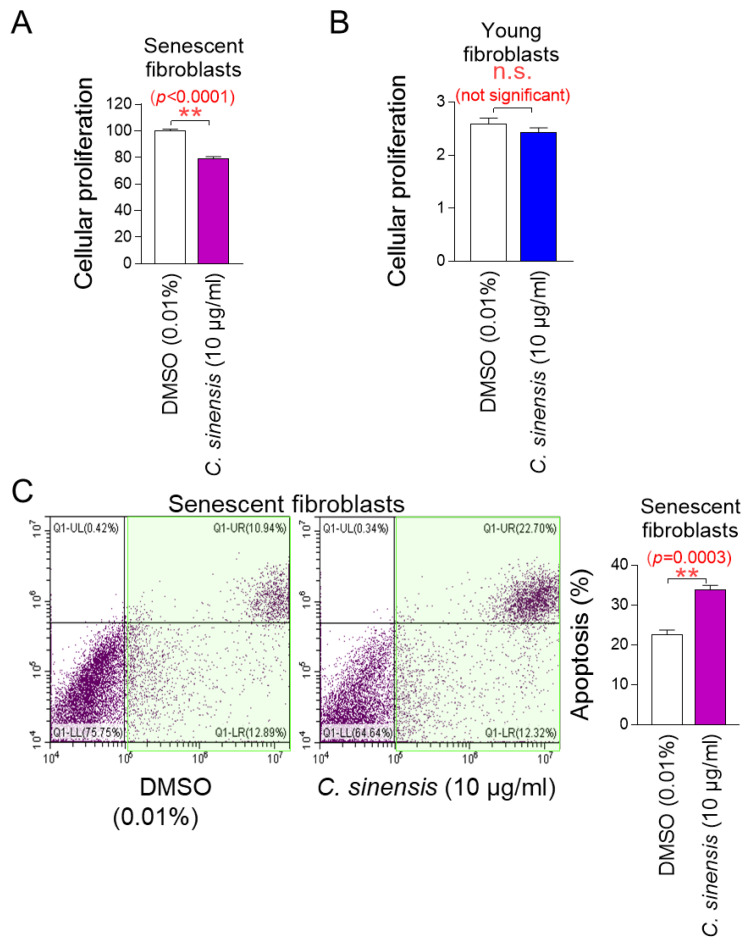
*C. sinensis* extract selectively kills senescent fibroblasts by inducing apoptosis. (**A**) Senescent fibroblasts were treated with dimethyl sulfoxide (DMSO) (0.01%) or *Camellia sinensis* (*C. sinensis*) extract (10 μg/mL) for 12 days. Then, cellular proliferation was evaluated. ** *p* < 0.01, Student’s *t*-test. Mean ± S.D., *n* = 6. (**B**) Young fibroblasts were treated with DMSO (0.01%) or *C. sinensis* extract (10 μg/mL) for 12 days. Then, cellular proliferation was evaluated. n.s. (not significant), Student’s *t*-test. Mean ± S.D., *n* = 6. (**C**) Senescent fibroblasts were treated with DMSO (0.01%) or *C. sinensis* extract (10 µg/mL) for 12 days. Then, flow cytometric analysis (FACS) of apoptosis was evaluated. The green square represents apoptotic cell populations. ** *p* < 0.01, Student’s *t*-test. Mean ± S.D., *n* = 3.

The determination of the optimal concentration of *C. sinensis* extract led to the investigation of whether *C. sinensis* extract acts as a senolytic that selectively induces the apoptosis of senescent cells or as a senomorphic that increases the proliferation of senescent cells. To investigate the role of *C. sinensis* extract, we measured the proliferation of senescent fibroblasts after treatment with *C. sinensis* extract. Treatment with *C. sinensis* extract significantly reduced the cell proliferation of senescent fibroblasts compared to DMSO control (Figure 2A).

Next, the proliferation of young fibroblasts after treatment with *C. sinensis* extract was examined. Treatment with *C. sinensis* extract for 12 days did not affect the proliferation of young fibroblasts (Figure 2B). These results suggest that the C. sinensis extract acts as senolytics by inhibiting the proliferation of senescent fibroblasts but not affecting non-senescent cells.

We then evaluated the apoptosis rate to determine whether the inhibition of proliferation of senescent fibroblasts by *C. sinensis* extract was due to apoptosis. Treatment with *C. sinensis* extract significantly increased the apoptosis rate of senescent fibroblasts compared to the DMSO control, confirming that *C. sinensis* extract acts as senolytics by inducing the apoptosis of senescent fibroblasts (Figure 2C).

### 2.3. C. sinensis Extract Decreases Mitochondrial ROS Production by Enhancing OXPHOS Efficiency

**Figure 3:**
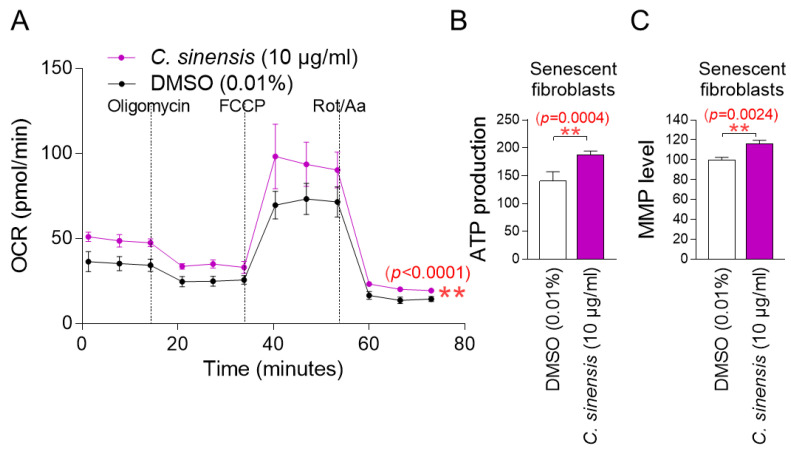
*C. sinensis* extract decreases mitochondrial ROS production by enhancing OXPHOS efficiency. (**A**) Senescent fibroblasts were treated with dimethyl sulfoxide (DMSO) (0.01%) or *Camellia sinensis* (*C. sinensis*) extract (10 μg/mL) for 12 days. Oxygen consumption rate (OCR; mpol/min) was measured (black line: DMSO–treated senescent fibroblasts, purple line: *C. sinensis* extract-treated senescent fibroblasts). ** *p* < 0.01, two-way ANOVA followed by Bonferroni’s post hoc test. Means ± S.D., *n* = 3. (**B**) Senescent fibroblasts were treated with DMSO (0.01%) or *C. sinensis* extract (10 µg/mL) for 12 days and their ATP production was measured. ** *p* < 0.01, Student’s *t*-test. Means ± S.D., *n* = 3. (**C**) Senescent fibroblasts were treated with DMSO (0.01%) or *C. sinensis* extract (10 µg/mL) for 12 days and mitochondrial membrane potential (MMP) was measured. ** *p* < 0.01, Student’s *t*-test. Mean ± S.D., *n* = 3.

The observation that *C. sinensis* extract acts as senolytics raises the question of how it reduces mitochondrial ROS generation. To understand the mechanism of mitochondrial ROS reduction by *C. sinensis* extract, OXPHOS efficiency was evaluated. The oxygen consumption rate (OCR; pmoles/min) was examined as a measure of OXPHOS efficiency [37]. The treatment included oligomycin, carbonyl cyanide-p-trifluoromethoxyphenylhydrazone (FCCP), and rotenone/antimycin A. ATP-coupled respiration (following oligomycin treatment), maximal respiration (following FCCP treatment), and non-mitochondrial respiration (following rotenone/antimycin A treatment) were assessed using OCR values. *C. sinensis* extract treatment significantly increased OCR values compared to DMSO control, demonstrating that *C. sinensis* extract increased increasing ATP-coupled respiration, maximal respiration, and non-mitochondrial respiration (Figure 3A; black vs. purple lines). These data indicate that *C. sinensis* extract enhances OXPHOS efficiency. Since increased OXPHOS efficiency indirectly indicates efficient electron transport in the ETC, these results suggest that efficient electron transport by *C. sinensis* extract acts as a novel mechanism to reduce mitochondrial ROS production.

The finding that *C. sinensis* extract increases OXPHOS efficiency led us to investigate whether this would lead to increased mitochondrial ATP production. Compared to the DMSO control, *C. sinensis* extract significantly increased ATP production (Figure 3B). This finding suggests that *C. sinensis* extract increased ATP synthesis by increasing the efficiency of OXPHOS.

Mitochondrial damage caused by ROS reduces mitochondrial membrane potential (MMP), which is generated when protons move from the matrix to the mitochondrial intermembrane space [38]. Since we found that *C. sinensis* extract reduced mitochondrial ROS levels, we investigated whether *C. sinensis* extract had any effect on MMP. Senescent fibroblasts treated with *C. sinensis* extract had a significant increase in MMP compared to fibroblasts treated with DMSO (Figure 3C). This finding indicates the *C. sinensis* extract-mediated mitochondrial functional recovery.

### 2.4. Senescence-Associated Phenotypes Are Ameliorated by C. sinensis Extract

**Figure 4:**
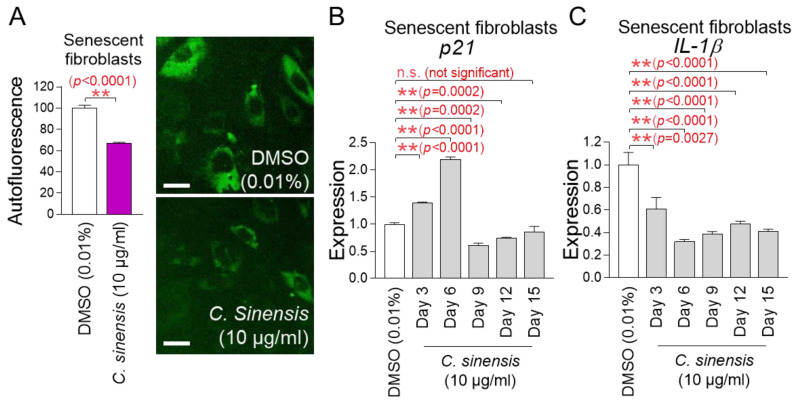
Senescence-associated phenotypes are ameliorated by *C. sinensis* extract. (**A**) Senescent fibroblasts were treated with dimethyl sulfoxide (DMSO) (0.01%) or *Camellia sinensis* (*C. sinensis*) extract (10 μg/mL) for 12 days. Autofluorescence was measured using FACS. ** *p* < 0.01, Student’s *t*-test. Mean ± S.D., *n* = 3. Autofluorescence (green) was also observed using a fluorescence microscope. Scale bar: 10 μm. (**B**,**C**) After 12 days of treatment with DMSO (0.01%) or *C. sinensis* extract (10 µg/mL) in senescent fibroblasts, expression levels of *p21* or *IL-1β* were evaluated. n.s. (not significant), ** *p* < 0.01, Student’s *t*-test. Mean ± S.D., *n* = 3.

Mitochondrial functional recovery serves as a precondition for improving senescence [39]. The finding that *C. sinensis* extract restores mitochondrial function led us to investigate its impact on senescence. Lipofuscin was re-measured by detecting intracellular autofluorescence [36]. Senescent fibroblasts treated with *C. sinensis* extract had a significant decrease in autofluorescence compared to fibroblasts treated with DMSO, demonstrating that *C. sinensis* extract improved one of the main characteristics of senescence (Figure 4A).

The p53/p21 pathway is a key regulatory mechanism for cell cycle arrest in the early stage of senescence [40]. Since p21 acts as a downstream signal of p53, we investigated the expression changes in p21. To further study the changes in *p21* expression, gene expression was measured on 3, 6, 9, 12, and 15 days after treatment with *C. sinensis* extract. Treatment with *C. sinensis* extract for 3 and 6 days significantly increased *p21* expression in senescent fibroblasts compared to the DMSO control (Figure 4B). However, treatment with *C. sinensis* extract for 9 and 12 days significantly decreased *p21* expression compared to the DMSO control (Figure 4B). Moreover, treatment with *C. sinensis* extract for 15 days subtly decreased *p21* expression compared to the DMSO control (Figure 4B). These data indicate that cell cycle arrest was alleviated from 9 days after treatment with *C. sinensis* extract.

Senescence-associated secretory phenotype (SASP) is defined as cytokines and chemokines produced by senescent cells [41]. ROS combine with mitochondrial superoxide dismutase in the matrix to generate hydrogen peroxide, which can cross the mitochondrial outer membrane and damage cytosolic proteins [42]. This reaction triggers the release of SASP (IL-1β, IL-6, IL-8, etc.) [43,44,45]. Among the SASP, IL-1β, known as an inflammatory SASP, was investigated [46]. To further study the changes in *IL-1β* expression, gene expression was measured on 3, 6, 9, 12, and 15 days after treatment with *C. sinensis* extract. Treatment with *C. sinensis* extract for 3, 6, 9, 12, and 15 days significantly decreased *IL-1β* expression in senescent fibroblasts compared to the DMSO control, thereby downregulating the inflammatory SASP (Figure 4C).

### 2.5. C. sinensis Extract Improves Skin Aging Through Collagen Synthesis and Remodeling

**Figure 5:**
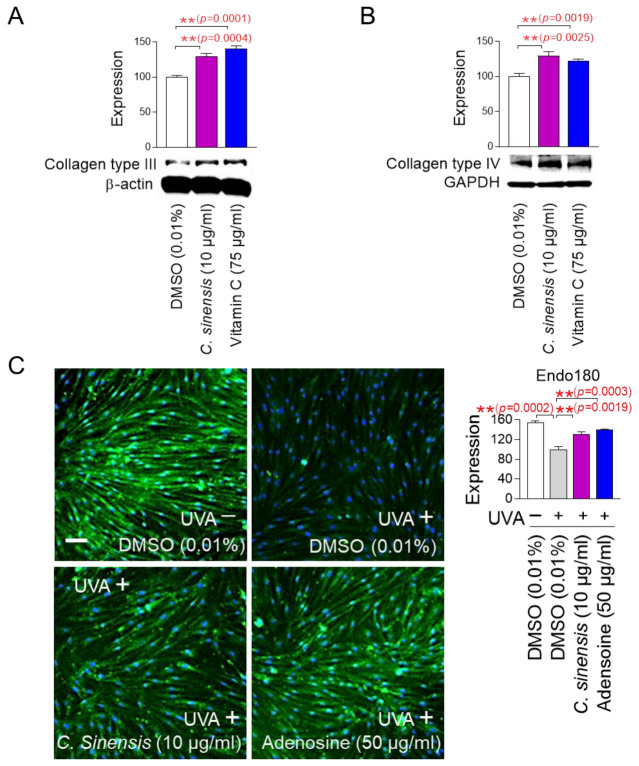
*C. sinensis* extract improves skin aging through collagen synthesis and remodeling. (**A**,**B**) After 12 days of treatment with dimethyl sulfoxide (DMSO) (0.01%), *Camellia sinensis* (*C. sinensis*) extract (10 µg/mL), or vitamin C (75 µg/mL) in senescent fibroblasts, expression levels of collagen type III and IV were evaluated. ** *p* < 0.01, Student’s *t*-test. Mean ± S.D., *n* = 3. (**C**) To inhibit the expression of endo 180, senescent fibroblasts were irradiated with 25 J/cm^3^ ultraviolet A (UVA). Then, senescent fibroblasts were treated with DMSO (0.01%), *C. sinensis* extract (10 µg/mL), or adenosine (50 μg/mL) for 1 day. ** *p* < 0.01, student *t*-test. Mean ± S.D., *n* = 3. Scale bar: 50 μm. Full-size images of immunofluorescence are shown in Supplementary Figure S1.

The finding that *C. sinensis* extract improves senescence-associated phenotypes led us to investigate how this extract affects skin aging. To investigate how *C. sinensis* extract affects skin aging, we investigated the changes in the expression patterns of each collagen type. Vitamin C, which helps in the redox recycling of other antioxidants, was used as a positive control [47]. *C. sinensis* extract, like vitamin C, significantly upregulated collagen type III expression compared to DMSO control, suggesting that *C. sinensis* extract was beneficial in skin regeneration (Figure 5A). *C. sinensis* extract. like vitamin C, also showed that it was effective in providing structural support to skin tissue by significantly increasing the expression of collagen type IV compared to DMSO control (Figure 5B).

In the dermal tissue, collagen fibers are maintained through a remodeling process consisting of synthesis and degradation [48]. The degradation process consists of the cleavage of collagen fibers by matrix metalloproteases and the internalization of collagen degradation products by endo180 [49]. Ultraviolet (UV) exposure induces photoaging by reducing the expression of endo180, thereby inhibiting the internalization of collagen degradation products [50]. To investigate the role of *C. sinensis* extract on endo180 expression, senescent fibroblasts were irradiated with UVA and then treated with *C. sinensis* extract. UVA irradiation significantly decreased endo180 expression compared to the non-UVA-irradiated group (Figure 5C and Figure S1). Adenosine, a known activator of collagen production, was used as a positive control [51]. Adenosine treatment significantly restored the decreased endo180 expression caused by UVA irradiation (Figure 5C and Figure S1). *C. sinensis* extract also significantly restored the decreased endo180 expression caused by UVA irradiation (Figure 5C and Figure S1). These results suggest that *C. sinensis* extract promotes efficient collagen remodeling by restoring endo180 expression.

### 2.6. C. sinensis Extract Improves Skin Aging Through Enhancing Cell-Induced Collagen Contractility

**Figure 6:**
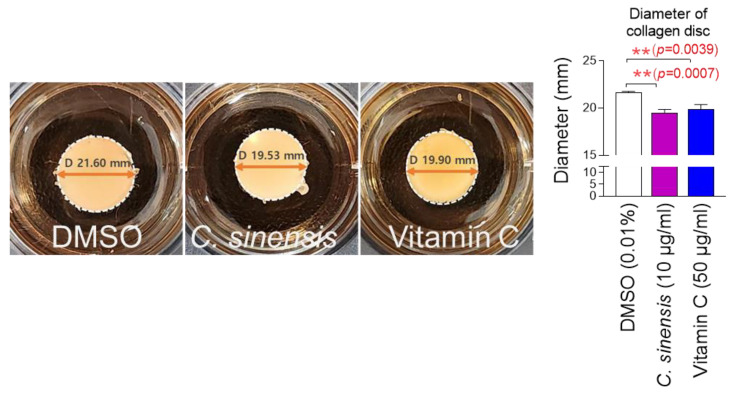
*C. sinensis* extract improves skin aging through enhancing cell-induced collagen contractility. Senescent fibroblasts embedded in collagen gels were treated with dimethyl sulfoxide (DMSO) (0.01%), *Camellia sinensis* (*C. sinensis*) extract (10 µg/mL), or vitamin C (50 μg/mL) for 1 day. The cell-induced contractility of collagen is evaluated by measuring the diameter of the collagen disc. ** *p* < 0.01, student *t*-test. Mean ± S.D., *n* = 3.

The finding that *C. sinensis* extract increases collagen synthesis and remodeling led us to investigate how this extract affects the cells’ ability to contract collagen. Collagen gel contraction assay is a method to quantify cell-induced collagen contractility [52]. Since cells embedded in a disc-shaped collagen gel contract collagen and reduce the diameter of the collagen disc, the diameter of the disc is used to measure cell-induced collagen contractility [52]. To investigate the role of *C. sinensis* extract on cell-induced collagen contractility, senescent fibroblasts embedded in collagen gels were treated with *C. sinensis* extract. Antioxidant vitamin C was used as a positive control [47]. Vitamin C significantly reduced the collagen gel diameter compared to DMSO control, indicating the increased cell-induced collagen contractility (Figure 6). *C. sinensis* extract also significantly reduced the collagen gel diameter compared to the DMSO control, suggesting that *C. sinensis* extract improves skin aging through enhancing cell-induced collagen contractility (Figure 6).

### 2.7. C. sinensis Extract Restores the Skin Barrier Function

**Figure 7:**
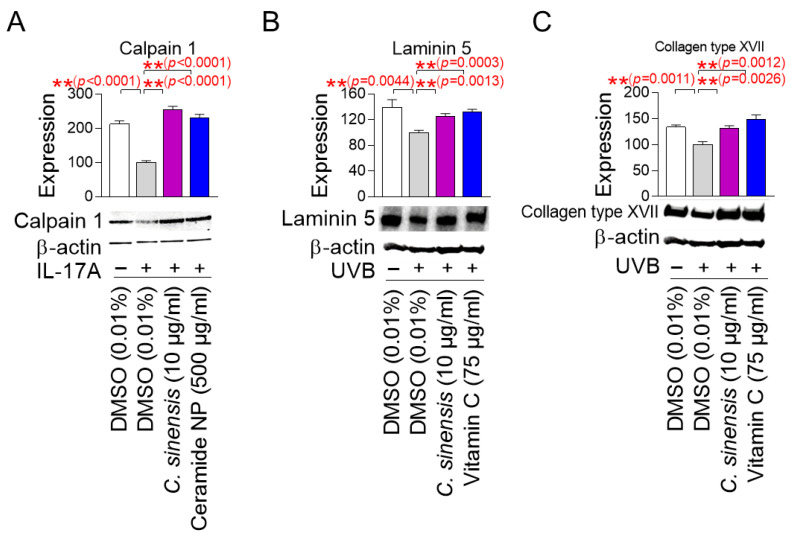
*C. sinensis* extract restores the skin barrier function. (**A**) To inhibit the expression of calpain 1, human epidermal keratinocyte (HEKn) cells were stimulated with 200 ng/mL interleukin-17A (IL-17A). Then, HEKn cells were treated with DMSO (0.01%), *C. sinensis* extract (10 µg/mL), or ceramide NP (500 μg/mL) for 1 day. ** *p* < 0.01, Student’s *t*-test. Mean ± S.D., *n* = 3. (**B**) To inhibit the expression of laminin 5, A431 cells were irradiated with 50 mJ/cm^3^ ultraviolet B (UVB). Then, A431 cells were treated with dimethyl sulfoxide (DMSO) (0.01%), *Camellia sinensis* (*C. sinensis*) extract (10 µg/mL), or vitamin C (75 μg/mL) for 1 day. ** *p* < 0.01, Student *t*-test. Mean ± S.D., *n* = 3. (**C**) To inhibit the expression of collagen type XVII, A431 cells were irradiated with 50 mJ/cm^3^ UVB. Then, A431 cells were treated with DMSO (0.01%), *C. sinensis* extract (10 µg/mL), or vitamin C (75 μg/mL) for 1 day. ** *p* < 0.01, student *t*-test. Mean ± S.D., *n* = 3.

Calpain 1 promotes epidermal barrier formation by inhibiting several inflammatory pathways [53,54]. In contrast, IL-17A induces skin inflammation by stimulating T cells to produce numerous inflammatory cytokines [53,54]. Since skin inflammation is generated by cytokines secreted when the stratum corneum of the epidermis is damaged [55], human epidermal keratinocytes, HEKn cells, which are very similar to the stratum corneum, have been widely used in skin inflammation studies [56,57]. To study the effect of *C. sinensis* extract on calpain 1 expression, HEKn cells were stimulated with IL-17A and then treated with *C. sinensis* extract. Ceramide NP was used as a positive control, because their structure is similar to the lipid barrier of the skin and has been shown to improve the lipid barrier of aged skin [58]. IL-17A significantly decreased the expression of calpain 1 protein compared to the non-IL-17A treated group, confirming the induction of skin inflammation by IL-17A (Figure 7A). However, ceramide NP significantly restored the decreased calpain 1 expression caused by IL-17A treatment (Figure 7A). Furthermore, *C. sinensis* extract significantly recovered the decreased calpain 1 expression caused by IL-17A treatment, indicating that *C. sinensis* extract restored the skin barrier function through inhibiting skin inflammation (Figure 7A).

Laminin 5 is located at the basement membrane of the dermal–epidermal junction (DEJ) and serves as an essential matrix protein connecting dermal and epithelial cells [59]. It acts as a bridge to connect keratinocytes with the collagen structure of the dermis to maintain the structural stability of the skin [59]. The DEJ acts as a barrier that protects the skin from the external environment, and laminin 5 is essential for maintaining the integrity of this barrier [60]. On the other hand, UVB irradiation damages the laminin integrity of skin cells at the DEJ [61]. Since A431 cells with an epithelial morphology have been widely used to study the protein expression level of laminin 5 [62,63], A431 cells were irradiated with UVB and then treated with *C. sinensis* extract. Vitamin C, which stimulates laminin production, was used as a positive control [64]. UVB irradiation significantly increased the expression of laminin 5 protein compared to the non-UVB-irradiated group, confirming the UVB-mediated decrease in laminin expression (Figure 7B). However, vitamin C significantly restored the decrease in laminin 5 expression caused by UVB exposure (Figure 7B). Furthermore, *C. sinensis* extract restored the decrease in laminin 5 expression caused by UVB irradiation. These results suggest that *C. sinensis* extract may help restore skin defense by enhancing the stability of DEJ.

Collagen XVII is located in the basement membrane of the DEJ and serves as a structural component that allows keratinocytes to attach to the basement membrane [65]. Collagen XVII is essential for maintaining the integrity of the skin barrier by anchoring the dermal layer and reinforcing the physical barrier function of the DEJ [66]. On the other hand, UV irradiation to human skin and keratinocytes reduces collagen type XVII expression [67]. To investigate the effect of *C. sinensis* extract on collagen type XVII expression, A431 cells were exposed to UVB and then treated with *C. sinensis* extract. Vitamin C, which plays an important role in collagen synthesis, was used as a positive control [68]. UVB irradiation significantly decreased collagen type XVII expression compared to the non-UVB-irradiated group (Figure 7C). However, vitamin C significantly restored the decreased collagen type XVII expression due to UVB irradiation (Figure 7C). Moreover, *C. sinensis* extract significantly recovered the decreased collagen type XVII expression due to UVB irradiation, indicating that it has a positive effect on skin barrier function through the recovery of DEJ function.

### 2.8. Identification of Epigallocatechin Gallate (EGCG) as the Active Ingredient in C. sinensis Extract

After finding that *C. sinensis* extract is effective in improving skin aging and restoring the skin barrier, we decided to investigate which components in *C. sinensis* extract are responsible for these effects. In previous study, epicatechin (EC), epicatechin gallate (ECG), and epigallocatechin gallate (EGCG) were identified as the major components of *C. sinensis* extract [69]. These compounds, classified as catechins, contain natural phenolic agents that act as oxygen radical scavengers through redox processes [70]. To determine which components are key to the antioxidant effects of *C. sinensis* extract, senescent fibroblasts were grown in culture medium containing various concentrations of EC, ECG, and EGCG. Then, the effect on mitochondrial ROS levels was investigated using DHR123. In addition, autofluorescence was measured to evaluate the subsequent effects on lipofuscin levels. *C. sinensis* extract was used as a positive control.

**Figure 8:**
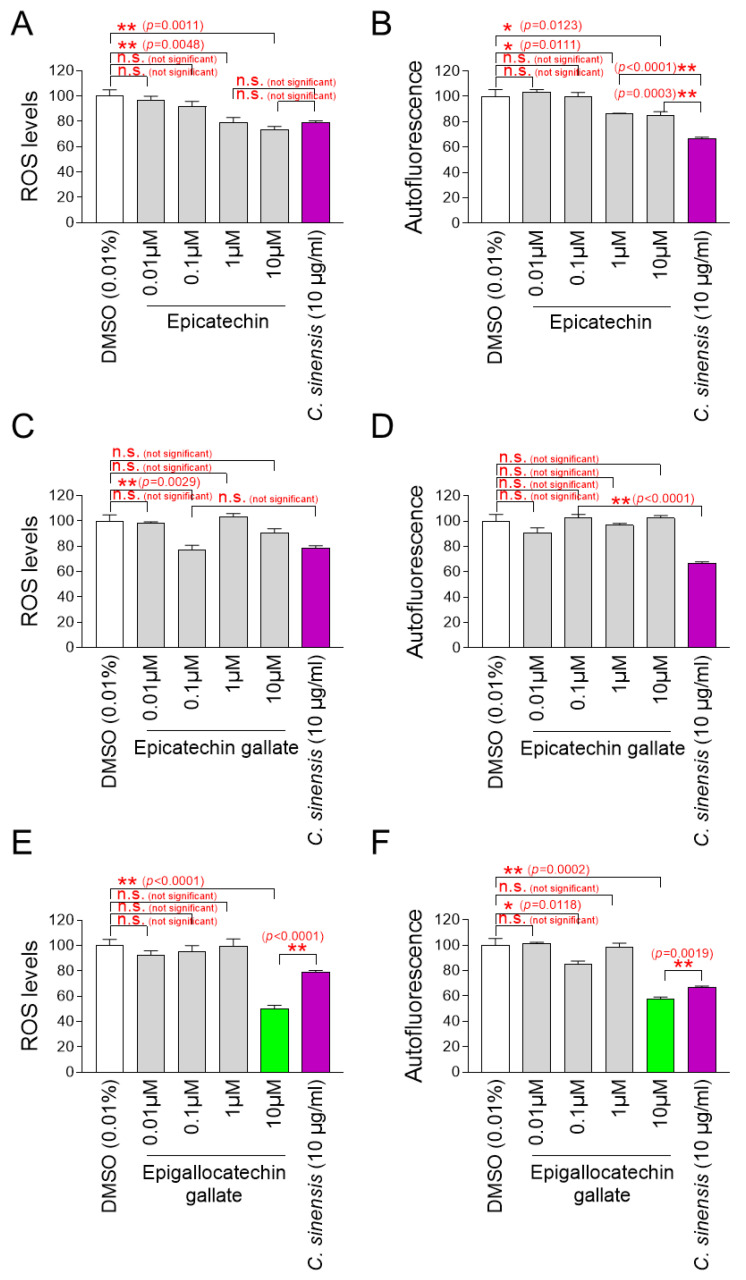
Identification of epigallocatechin gallate (EGCG) as the active ingredient in *C. sinensis* extract. (**A**,**B**) Senescent fibroblasts were treated with dimethyl sulfoxide (DMSO) (0.01%), epicatechin (0.01, 0.1, 1, and 10 µM), or *Camellia sinensis* (*C. sinensis*) extract (10 µg/mL) for 12 days. Mitochondrial ROS levels using dihydrorhodamine 123 (DHR123) and autofluorescence were evaluated. n.s. (not significant), * *p* < 0.05, ** *p* < 0.01, Student’s *t*-test. Mean ± S.D., *n* = 3. (**C**,**D**) Senescent fibroblasts were treated with DMSO (0.01%), epicatechin gallate (0.01, 0.1, 1, and 10 µM), or *C. sinensis* extract (10 µg/mL) for 12 days. Mitochondrial ROS levels using DHR123 and autofluorescence were evaluated. n.s. (not significant), ** *p* < 0.01, Student’s *t*-test. Mean ± S.D., *n* = 3. (**E**,**F**) Senescent fibroblasts were treated with DMSO (0.01%), epigallocatechin gallate (0.01, 0.1, 1, and 10 µM), or *C. sinensis* extract (10 µg/mL) for 12 days served as a positive control. Mitochondrial ROS levels using DHR123 and autofluorescence were evaluated. n.s. (not significant), * *p* < 0.05, ** *p* < 0.01, Student’s *t*–test. Mean ± S.D., *n* = 3.

A significant reduction in mitochondrial ROS and autofluorescence was observed at ECs at 1 and 10 μM compared to DMSO control (Figure 8A,B). However, compared to *C. sinensis* extract, ECs at 1 and 10 μM were not more effective in reducing mitochondrial ROS and autofluorescence (Figure 8A,B).

Mitochondrial ROS was significantly reduced at 0.1 μM ECG, but autofluorescence was not significantly reduced at all ECG concentrations (Figure 8C,D). Compared to *C. sinensis* extract, ECG at 0.1 μM was not more effective in reducing mitochondrial ROS (Figure 8C).

While EGCG at 0.1 μM did not significantly reduce mitochondrial ROS compared to DMSO control, EGCG at 0.1 μM significantly reduced autofluorescence (Figure 8E,F). However, EGCG at 10 μM significantly reduced both mitochondrial ROS and autofluorescence compared to DMSO control (Figure 8E,F). Compared to the *C. sinensis* extract, EGCG at 10 μM was more effective in reducing mitochondrial ROS and autofluorescence (Figure 8E,F).

Based on these findings, EGCG was chosen as an active ingredient that has antioxidant and anti-aging properties. The chosen concentration for the following studies was 10 μM of EGCG.

### 2.9. EGCG, the Active Ingredient in C. sinensis Extract, Exhibits Similar Effects to C. sinensis Extract

**Figure 9:**
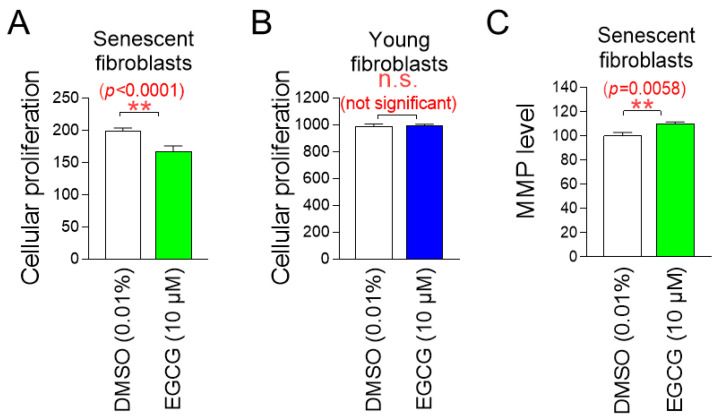
EGCG, the active ingredient in *C. sinensis* extract, exhibits similar effects to *C. sinensis* extract. (**A**) Senescent fibroblasts were treated with dimethyl sulfoxide (DMSO) (0.01%) or epigallocatechin gallate (EGCG) (10 µM) for 9 days. Then, cellular proliferation was evaluated. ** *p* < 0.01, Student’s *t*-test. Mean ± S.D., *n* = 6. (**B**) Young fibroblasts were treated with DMSO (0.01%) or EGCG (10 µM) for 9 days. Then, cellular proliferation was evaluated n.s. (not significant), Student’s *t*-test. Mean ± S.D., *n* = 6. (**C**) Senescent fibroblasts were treated with DMSO (0.01%) or EGCG (10 µM) for 9 days. Then, mitochondrial membrane potential (MMP) was measured. ** *p* < 0.01, Student’s *t*-test. Mean ± S.D., *n* = 3.

Having discovered that EGCG is the active ingredient in *C. sinensis* extract, we investigated the effects of EGCG on cell proliferation in senescent and young fibroblasts to determine whether it has a similar role as *C. sinensis* extract. EGCG significantly inhibited the cell proliferation of senescent fibroblasts, whereas EGCG did not inhibit cell proliferation of young fibroblasts (Figure 9A,B). These data indicate that EGCG selectively inhibits the proliferation of senescent fibroblasts without affecting young fibroblasts.

We then examined the effects of EGCG on mitochondrial function, specifically MMP. Senescent fibroblasts treated with EGCG showed a significant increase in MMP compared to fibroblasts treated with DMSO (Figure 9C). These data indicate that EGCG induces the restoration of mitochondrial function similar to *C. sinensis* extract.

### 2.10. C. Sinensis Extracts and EGCG Reverse Skin Aging in Artificial Skin Models

**Figure 10:**
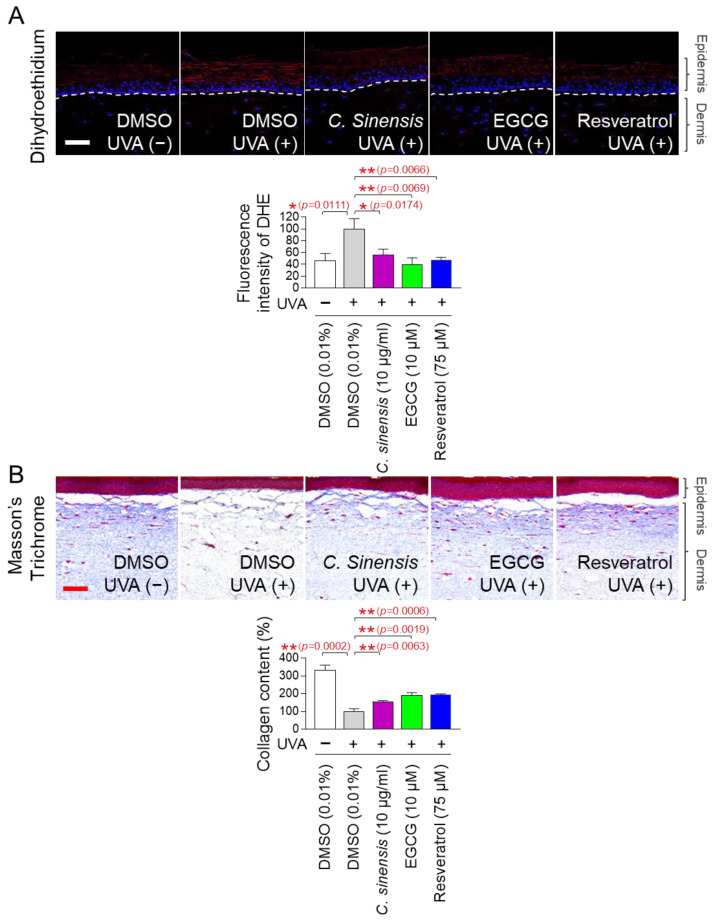
*C. sinensis* extracts and EGCG reverse skin aging in artificial skin models. (**A**,**B**) To induce skin aging, artificial skin models were irradiated with 20 mJ/cm^3^ ultraviolet A (UVA) once a day for three times. Then, artificial skin models were treated with dimethyl sulfoxide (DMSO) (0.01%), *Camellia sinensis* (*C. sinensis*) extract (10 µg/mL), epigallocatechin gallate (EGCG) (10 µM), or resveratrol (75 μM) for 1 day. To detect ROS in artificial skin models, dihydroethidium (DHE) staining was performed. To distinguish collagen fibers from other tissues, Masson’s trichrome (MT) staining was performed. Scale bar: 10 μm. * *p* < 0.05, ** *p* < 0.01, Student’s *t*-test. Mean ± S.D., *n* = 3.

The discovery of anti-aging effects of *C. sinensis* extract and EGCG at the cellular level led us to investigate whether these effects were also observed at the skin tissue level. An artificial skin model composed of a functional multilayer epidermis similar to human epidermis was used [71,72]. First, the effects of *C. sinensis* extract and EGCG on ROS levels were investigated using dehydroethidium (DHE) staining. DHE reacts with O_2_^●–^ to produce red fluorescence and was used to detect ROS in skin tissue [73]. Resveratrol served as a positive control [34]. UVA irradiation significantly increased the red fluorescence level in the artificial skin model compared to the non-UVA-irradiated group (Figure 10A). However, resveratrol significantly reduced the red fluorescence increased by UVA irradiation (Figure 10A). Similarly, *C. sinensis* extract and EGCG also significantly reduced the red fluorescence increased by UVA irradiation (Figure 10A).

Based on the results that *C. sinensis* extract and EGCG effectively reduced ROS in the artificial skin model, we investigated whether they could improve skin aging through collagen synthesis. Masson’s trichrome (MT) staining is a widely used technique for visualizing connective tissue in tissue sections [74]. This method is particularly used to distinguish collagen fibers from other tissues by staining collagen fibers in blue [74]. After UVA treatment to induce skin aging in the artificial skin model, we investigated the effects of *C. sinensis* extract and EGCG on collagen synthesis through MT staining. Resveratrol served as a positive control [34]. UVA irradiation significantly decreased collagen content compared to the non-UVA irradiation group (Figure 10B). However, resveratrol significantly restored the collagen content decreased by UVA irradiation (Figure 10B). The groups treated with *C. sinensis* extract and EGCG also significantly restored the collagen content decreased by UVA irradiation (Figure 10B). These data show that *C. sinensis* extract and EGCG reverse skin aging by increasing collagen synthesis.

## 3. Discussion

Senotherapy for treating aging consists of senolytics and senomorphics [75]. Senolytics selectively remove senescent cells that can impair the function of normal cells [19]. Senomorphics change the phenotype of senescent cells to that of young cells by modulating various signaling pathways [75]. Senomorphics, which transform senescent cells with cell cycle arrest characteristics into young cells with high proliferative potential, may be effective in reversing aging but may have potential side effects due to their similarity to tumorigenesis mechanisms [76]. However, senolytics are emerging as a therapeutic strategy that can minimize these side effects, because they selectively remove senescent cells [77]. Quercetin, which inhibits B-cell lymphoma 2 (BCL2), a cell signaling pathway that blocks programmed cell death, is one of the most widely used senolytics [21]. Quercetin significantly decreased SA-β-gal-positive cells and inflammatory cytokines by selectively killing senescent cells [21]. Another inhibitor of the BCL2 pathway, navitoclax, effectively eliminated senescent cells by activating p53 [78]. When navitoclax was administered orally to mildly irradiated or normally aged mice, senescent cells were reduced [78]. Despite the beneficial effects of senolytics on senescence, their use has been limited by findings showing that eliminating senescent cells may simultaneously impair the overall regenerative capacity of the organism, leading to a more rapid accumulation of senescent cells [79]. Strategies are needed to identify novel senolytics that selectively kill senescent cells without impairing normal regenerative capacity. In this study, we screened substances that inhibit mitochondrial ROS and discovered *C. sinensis* extract as a candidate. *C. sinensis* extract selectively eliminated senescent fibroblasts through apoptosis without impairing regenerative capacity, as shown by the restored skin aging and skin barrier. Moreover, the regenerative capacity was proven not only at the cellular level but also at the skin tissue level, further increasing the value of *C. sinensis* extract as senolytics. Here, we propose a novel role for *C. sinensis* extract as senolytics that selectively removes senescent fibroblasts to improve skin aging.

The progression of senescence is closely related to damage to cellular organelles due to ROS-mediated oxidative stress [8]. Mitochondria are the main organelles that generate ROS [8]. Mitochondrial ETC converts 1–5% of the oxygen consumed in mitochondria into O_2_^●−^ [10]. In particular, oxygen is changed into O_2_^●−^ by complexes I and III in the mitochondrial matrix. In the intermembrane space of the mitochondria, oxygen is changed into O_2_^●−^ by complexes III. Senescence leads to mitochondrial deterioration, which reduces the activities of mitochondrial ETC [80,81]. The impaired complex I function results in inefficient electron transport, which leaks electrons to oxygen and generates O_2_^●−^ [82]. Increased ROS production in mitochondria aggravates ETC damage, which further increases ROS production in mitochondria [11]. This harmful cycle impairs the composition and function of cellular organelles, ultimately accelerating senescence [83]. This causal link highlights the importance of lowering mitochondrial ROS production as a means of reversing aging [11]. In this study, we found the selective apoptosis of senescent fibroblasts by *C. sinensis* extract. During this process, senescent fibroblasts with dysfunctional mitochondria were eliminated, while non-senescent fibroblasts with functional mitochondria were maintained, which may have reduced mitochondrial ROS generation. However, we acknowledge that further studies are needed to prove this. In addition, we identified a novel mechanism in which *C. sinensis* extract reduces ROS production in mitochondria by increasing OXPHOS efficiency. The enhanced OXPHOS by *C. sinensis* extract suggests the restoration of impaired ETC function. Effective electron transport lowers electron leakage in mitochondria ETC, which lowers mitochondrial ROS generation as a byproduct [84]. The restoration of ETC function also increased MMP, which in turn increased ATP production, suggesting mitochondrial functional recovery. Furthermore, mitochondrial functional recovery by *C. sinensis* extract led to the improvement of various senescence-associated phenotypes. In conclusion, our study results demonstrate that *C. sinensis* extract can reduce ROS production in mitochondria, restore mitochondrial function, and ultimately, improve senescence-related phenotypes.

Natural substances are often used as cosmetic ingredients, because they are less likely to have negative effects on the skin than synthetically produced substances [85]. For example, aloe vera is known for its soothing and moisturizing effects and is used as an additive in a variety of cosmetics, especially moisturizers and soothing creams [86]. Similarly, chamomile is well-known for its soothing effects, and chamomile extracts are often included in creams for sensitive skin [87]. However, since natural products contain a variety of ingredients, it is an important process to identify active ingredients that are effective in improving skin aging. The discovery of active ingredients can eliminate unnecessary ingredients present in natural products as cosmetic ingredients, thereby preventing harmful effects that may occur due to unnecessary ingredients [88]. In addition, the composition of natural substances can change depending on factors such as cultivation location and climate [89]. Therefore, cosmetics that use natural substances may have different performances due to changes in the composition of the natural substance [90,91]. In this study, we found that EGCG is the active ingredient of *C. sinensis* extract. EGCG is a type of catechin, which contains polyphenol. Similar to *C. sinensis* extract, EGCG significantly reduced mitochondrial ROS levels in senescent fibroblasts, and this effect was carried over to the artificial skin model to reduce ROS. Furthermore, as shown in *C. sinensis* extract, EGCG improved senescence-associated phenotypes in senescent fibroblasts, and this effect was carried over to the artificial skin model to improve skin aging via collagen synthesis. To the best of our knowledge, this is the first study to demonstrate that EGCG, the active ingredient in *C. sinensis* extract, improves the phenotypes of senescent fibroblasts and aged skin. We propose that EGCG could be used as a cosmetic ingredient to effectively improve skin aging, while minimizing the adverse effects associated with the use of natural substances.

*C. sinensis* extract has been shown to improve skin aging by enhancing collagen synthesis and remodeling. Like *C. sinensis* extract, EGCG also showed an effect of improving skin aging through collagen production in an artificial skin model. However, further research is needed to directly use *C. sinensis* extract or EGCG as cosmetic ingredients. First, the efficacy of *C. sinensis* extract or EGCG was detected at 10 μg/mL, but a concentration optimization process is needed for cosmetic use. Serial dilutions of the compounds should be performed to evaluate the lowest concentration that exhibits skin improvement effects without toxicity [92,93]. Second, the extent of skin penetration by *C. sinensis* extract or EGCG should be determined. Only very small molecules and lipophilic substances can pass through the stratum corneum, which acts as a barrier [94]. If cosmetic ingredients cannot pass through the skin barrier, they will not be absorbed into the skin and will not be effective [95]. The skin penetration of *C. sinensis* extract or EGCG has not yet been investigated, but it can be improved by modifying their chemical groups [96]. In addition, the use of carriers that can pass through the skin barrier can improve skin penetration. For example, lipid nanoparticles or phospholipids can efficiently transport substances that are difficult to pass through the skin barrier [97]. Finally, more studies are needed to determine whether *C. sinensis* extract or EGCG interact with other substances to produce hazardous chemicals. Harmful chemicals, such as nitrosamines, can be formed when cosmetic ingredients combine with each other [98]. Therefore, in-depth studies should be performed to identify whether harmful substances are formed when *C. sinensis* extract or EGCG are used in combinations with other cosmetic compounds.

In summary, we found *C. sinensis* extract as a substance that reduces mitochondrial ROS production in senescent fibroblasts. Moreover, we discovered a novel mechanism by which *C. sinensis* extract reduces mitochondrial ROS production through selective apoptosis and enhanced OXPHOS efficiency in senescent fibroblasts. The novel mechanism of *C. sinensis* extract restores skin aging and the skin barrier by mitochondrial functional recovery. Furthermore, we discovered EGCG from *C. sinensis* extract as the active ingredient responsible for these beneficial effects. Similar to *C. sinensis* extract, EGCG reduced ROS and reversed skin aging in an artificial skin model. Our results suggest that *C. sinensis* extract or EGCG may be clinically useful for improving skin aging and the skin barrier.

## 4. Materials and Methods

### 4.1. Cell Culture

Human dermal fibroblasts (fibroblasts; PCS-201-010; ATCC, Manassas, VA, USA), human epidermal keratinocytes (HEKn; C0015C; Invitrogen, Waltham, MA, USA), and human epidermoid carcinoma (A431; CRL-1555; ATCC, Manassas, VA, USA) were used in this study. Human dermal fibroblasts were classified into senescent and young fibroblasts based on whether the cell population doubled in time greater than 14 days or less than 2 days. Human dermal fibroblasts and A431 cells were maintained in Dulbecco’s modified Eagle’s medium (DMEM; 10-013-cvc; CORNING, Corning, NY, USA) supplemented with 10% fetal bovine serum (FBS; 35-015-CV; CORNING, Corning, NY, USA) and 1% penicillin/streptomycin (15140-122; Gibco, Waltham, MA, USA). HEKn cells were cultured according to the method described in a previous study [99].

### 4.2. Preparation of Extract Powder

*C. sinensis* (Hadong, Gyeongsangnam-do, Republic of Korea) was mixed with deionized water in a volume ratio of 1:20 and heated at 80 °C for 5 h. After initial filtration through a mesh, an additional 5 µm and 0.5 µm filters were applied. The filtrate was completely concentrated using a vacuum evaporator and dried in a vacuum dryer (OV-12; JEIOTECH, Daejeon, Republic of Korea). *C. sinensis* extracts were diluted with dimethyl sulfoxide (DMSO, D8418; Sigma, St. Louis, MO, USA) to a concentration of 100 mg/mL. To obtain concentrations of 10 μg/mL of *C. sinensis* extracts, 1 μL of 100 mg/mL of *C. sinensis* extracts was added to 10 mL of medium. The DMSO control was created by diluting DMSO in the medium to a concentration of 0.01%.

### 4.3. Flow Cytometric Analysis of Reactive Oxygen Species (ROS), Mitochondrial Mass, and Lipofuscin

Senescent fibroblasts were administered with DMSO (0.01%), *C. sinensis* extract (10 μg/mL), or resveratrol (100 μM; 76511; Sigma, St. Louis, MO, USA) at 4-day intervals for 3, 6, 9, 12, and 15 days. To quantify mitochondrial ROS, cells were treated for 30 min at 37 °C in medium containing 5 μM dihydrorhodamine 123 (DHR123; D23806; Thermo Fisher Scientific, Waltham, MA, USA). In order to measure mitochondrial mass, cells were stained for 30 min at 37 °C in a medium containing 50 nM Mito-TrackerTM Deep Red FM Dye (M46753; Invitrogen, Waltham, MA, USA). In order to measure autofluorescence, cells were stained in dye-free media for 30 min at 37 °C. After that, FACS analysis was carried out as previously mentioned [100].

### 4.4. Cell Proliferation Assay

Senescent and young fibroblasts were cultivated in 96-well plates with a density of 1 × 10^3^ cells per well. Cells were administered with DMSO (0.01%) or *C. sinensis* extract (10 µg/mL) at 4-day intervals for 12 days. The EZ-cytox reagent (EZ-5000; DoGenBio, Seoul, Republic of Korea) was used according to manufacturer’s instructions.

### 4.5. Determination of Cell Viability

Senescent fibroblasts were administered with DMSO (0.01%) or *C. sinensis* extract (10 µg/mL) at 4-day intervals for 12 days. Cell viability was measured by trypan blue staining using Cedex HiRes analyzer (05650216001; Roche, Basel, Switzerland) [101]. Brightfield cell images were automatically captured on the Cedex HiRes analyzer. Cell viability was measured using digital image recognition technology in the Cedex HiRes analyzer [101].

### 4.6. Apoptosis Assay

Senescent fibroblasts were administered with DMSO (0.01%) or *C. sinensis* extract (10 µg/mL) at 4-day intervals for 12 days. The FITC Annexin V Apoptosis Detection Kit (556547; BD Biosciences, Franklin Lakes, NJ, USA) was utilized following the manufacturer’s instructions.

### 4.7. Measurement of Oxygen Consumption Rate (OCR)

Senescent fibroblasts were administered with DMSO (0.01%) or *C. sinensis* extract (10 µg/mL) at 4-day intervals for 12 days. The oxygen consumption rate (OCR) and ATP production rate were analyzed using a Seahorse Bioscience XFe24 flux analyzer (Billerica, MA, USA), as previously described [102]. Briefly, 5 × 10^4^ cells were seeded per well of XFe24 cell culture plates in XF24 FluxPak (100850-001; Seahorse Bioscience, Billerica, MA, USA). Cells were incubated at 37 °C in an incubator with 5% CO_2_ for 16 h. The medium was then changed to Seahorse Bioscience XF Assay medium (102365-100; Seahorse Bioscience, Billerica, MA, USA) without glucose. Cells were incubated for an additional 1 h in the same incubator. OCR and ATP production rates were determined using the Seahorse Bioscience XF Cell Mito Stress Test Kit (101706-100; Seahorse Bioscience, Billerica, MA, USA).

### 4.8. Measurement of Mitochondrial Membrane Potential (MMP) and Lipofuscin

Senescent fibroblasts were administered with DMSO (0.01%) or *C. sinensis* extract (10 µg/mL) at 4-day intervals for 12 days. To measure MMP, cells were treated for 30 min at 37 °C in medium containing 0.6 µg/mL JC-10 (ENZ-52305; Enzo Life Sciences, Farmingdale, NY, USA). Autofluorescence was measured by treating senescent fibroblasts in medium without dye for 30 min at 37 °C. Cells were analyzed by FACS, as previously described [103].

### 4.9. Quantitative Polymerase Chain Reaction (qPCR)

qPCR using each primer set was performed as previously described [104] (Table 1). In short, a CFX ConnectTM Real-Time PCR Detection System (Bio-Rad, Hercules, CA, USA) was used to perform qPCR utilizing Solg™ 2× Real-Time PCR Smart Mix (SRH83-M40h; Solgent, Daejeon, South Korea). After four minutes of denaturation at 95 °C, 40 cycles of 94 °C for 30 s, 57 °C for 30 s, and 70 °C for 10 s were carried out for qPCR.

### 4.10. Analysis of Collagen Synthesis

Senescent fibroblasts were administered with DMSO (0.01%), *C. sinensis* extract (10 µg/mL), or vitamin C (75 µg/mL; A4544; Sigma, St. Louis, MO, USA) at 4-day intervals for 12 days. Expression levels of collagen types III and IV were assessed by Western blot.

### 4.11. Analysis of Collagen Remodeling

To reduce endo180 protein expression, senescent fibroblasts were irradiated with 25 J/cm^3^ UVA. Then, senescent fibroblasts were treated with DMSO (0.01%), *C. sinensis* extract (10 µg/mL), or adenosine (50 µg/mL; A9251; Sigma, St. Louis, MO, USA) for 1 day. Immunofluorescence was performed following a previously established method [102]. Antibodies used were anti-endo180 (1:200 dilution, ab70132; Abcam, Cambridge, UK) and Alexa Fluor^®^ 488 goat anti-rabbit IgG antibody (1:200 dilution; A11008; Invitrogen, Waltham, MA, USA). Quantitative analysis was performed using Image J (https://imagej.net/ij/; National Institutes of Health (NIH), Bethesda, MD, USA).

### 4.12. Analysis of Cell-Induced Contractility of Collagen

To generate senescent fibroblasts embedded in collagen disc-shaped gels, the previous method was followed [105]. Senescent fibroblasts embedded in collagen gels were treated with DMSO (0.01%), *C. sinensis* extract (10 µg/mL), and vitamin C (50 µg/mL) for 1 day. The diameter of collagen discs was employed to determine cell-induced contractility. Image J (NIH, Bethesda, MD, USA) was used to carry out quantitative analysis.

### 4.13. Western Blot Analysis

Western blot analysis was performed, as previously described [106]. Briefly, cells were lysed in Laemmli sample buffer (1610737EDU; Bio-Rad, Hercules, CA, USA) containing 5% β-mercaptoethanol and heated at 100 °C for 5 min. The protein lysates were then separated on 10% Tris-glycine gels (4561033; Bio-Rad, Hercules, CA, USA). The separated proteins were transferred to polyvinylidene difluoride membranes (170-4156; Bio-Rad, Hercules, CA, USA) using a semidry apparatus (Bio-Rad, Hercules, CA, USA). The membranes were blocked with 5% nonfat dry milk in Tris-buffered saline containing 0.1% Tween. The following antibodies were used in this study: collagen type III antibody (1:1000 dilution; ab7778; Abcam, Cambridge, UK), collagen type IV antibody (1:1000 dilution; ab6586; Abcam, Cambridge, UK), calpain 1 antibody (1:1000 dilution; ab28258; Abcam, Cambridge, UK), laminin 5 antibody (1:1000 dilution; 13587; Santa cruz biotechnology, Dallas, TX, USA), collagen type XVII antibody (1:1000 dilution; ab184996; Abcam, Cambridge, UK), GAPDH antibody (1:1000 dilution; ab8245; Abcam), and β-actin antibody (1:5000 dilution; sc-47778; Santa cruz biotechnology, Dallas, TX, USA), horseradish peroxidase (HRP)-conjugated secondary antibody (1:2000 dilution; 1706515; Bio-Rad, Hercules, CA, USA), and HRP-conjugated secondary antibody (1:10,000 dilution; 1706516; Bio-Rad, Hercules, CA, USA).

### 4.14. Measurement of Calpain 1 Protein Expression

To inhibit calpain 1 expression, HEKn cells were treated with 200 ng/mL IL-17A (210-17; Peprotech, Cranbury, NJ, USA) for 1 day. Then, HEKn cells were treated with DMSO (0.01%), *C. sinensis* extract (10 µg/mL), or ceramide NPs (500 µg/mL; Ecotech, Gyeonggi-do, Republic of Korea) for 1 day. The expression levels of calpain 1 protein were evaluated using Western blot analysis.

### 4.15. Dermal–Epidermal Junction (DEJ) Analysis

To inhibit the expression of laminin 5 and collagen type XVII, A431 cells were irradiated with 50 mJ/cm^3^ UVB. Then, A431 cells were treated with DMSO (0.01%), *C. sinensis* extract (10 µg/mL), or vitamin C (75 µg/mL) for 1 day. The expression levels of laminin 5 and collagen type XVII proteins were measured by Western blot analysis.

### 4.16. Dehydroethidium (DHE) and Masson’s Trichrome (MT) Staining

To simulate skin aging, an artificial skin model (KeraSkin-FT^TM^; Biosolution, Seoul, Republic of Korea) was irradiated with 20 mJ/cm3 UVA once daily for 3 days. Then, the artificial skin models were treated with DMSO (0.01%), *C. sinensis* extract (10 µg/mL), EGCG (10 µM), or reserveratol (75 µM) for 1 day. To detect ROS in the artificial skin models, a DHE assay kit (ab236206; Abcam, Cambridge, UK) was used according to the manufacturer’s instructions. To distinguish collagen fibers from other tissues, an MT staining kit (ab150686; Abcam, Cambridge, UK) was used according to the manufacturer’s instructions.

### 4.17. Statistical Analysis

The Student’s *t*-tests was calculated using statistical software (GraphPad Prism 9; San Diego, CA, USA). Student’s *t*-test assumes that the sample population is normally distributed and has homogeneous variance. Two-way ANOVA followed by Bonferroni’s post-hoc was also calculated using statistical software (GraphPad Prism 9; San Diego, CA, USA).

## Figures and Tables

**Table 1:** List of primers.

Target	Orientation	Sequence (5′–3′)	Size (bp)
*36B4* (Accession number: NM_053275)	Forward	CAGCAAGTGGGAAGGTGTAATCC	23
	Reverse	CCCATTCTATCATCAACGGGTACAA	25
*p21* (Accession number: NM_000077.5)	Forward	AGGTGGACCTGGAGACTCTCAG	22
	Reverse	TCCTCTTGGAGAAGATCAGCCG	22
*IL-1β* (Accession number: NM_000576.3)	Forward	CCACAGACCTTCCAGGAGAATG	22
	Reverse	GTGCAGTTCAGTGATCGTACAGG	23

## Data Availability

The original contributions presented in the study are included in the article; further inquiries can be directed to the corresponding authors.

## Supplementary Materials

The following supporting information can be downloaded at https://www.mdpi.com/article/10.3390/ph18050612/s1, Figure S1. Full-size image of immunofluorescence in Figure 5C. (A) Original image of DMSO (UVA−) in Figure 5C. (B) Original image of DMSO (UVA+) in Figure 5C. (C) Original image of C. sinensis (UVA+) in Figure 5C. (D) Original image of adenosine (UVA+) in Figure 5C. Figure S2. Full-size image of Western blot. (A) Full-size image in Figure 5A. (B) Full-size image in Figure 5B. (C) Full-size image in Figure 7A. (D) Full-size image in Figure 7B. (E) Full-size image in Figure 7C.
