# Supplementary material for: Epigallocatechin Gallate in Camellia sinensis Ameliorates Skin Aging by Reducing Mitochondrial ROS Production
Source: Pharmaceuticals (Basel). 2025 Apr 23;18(5):612. doi: 10.3390/ph18050612 (PMC12114381; doi:10.3390/ph18050612)
Supplement: Supplementary file 1 [file pharmaceuticals-18-00612-s001.zip › pharmaceuticals-3556364-supplementary.pdf]

Supplementary Information

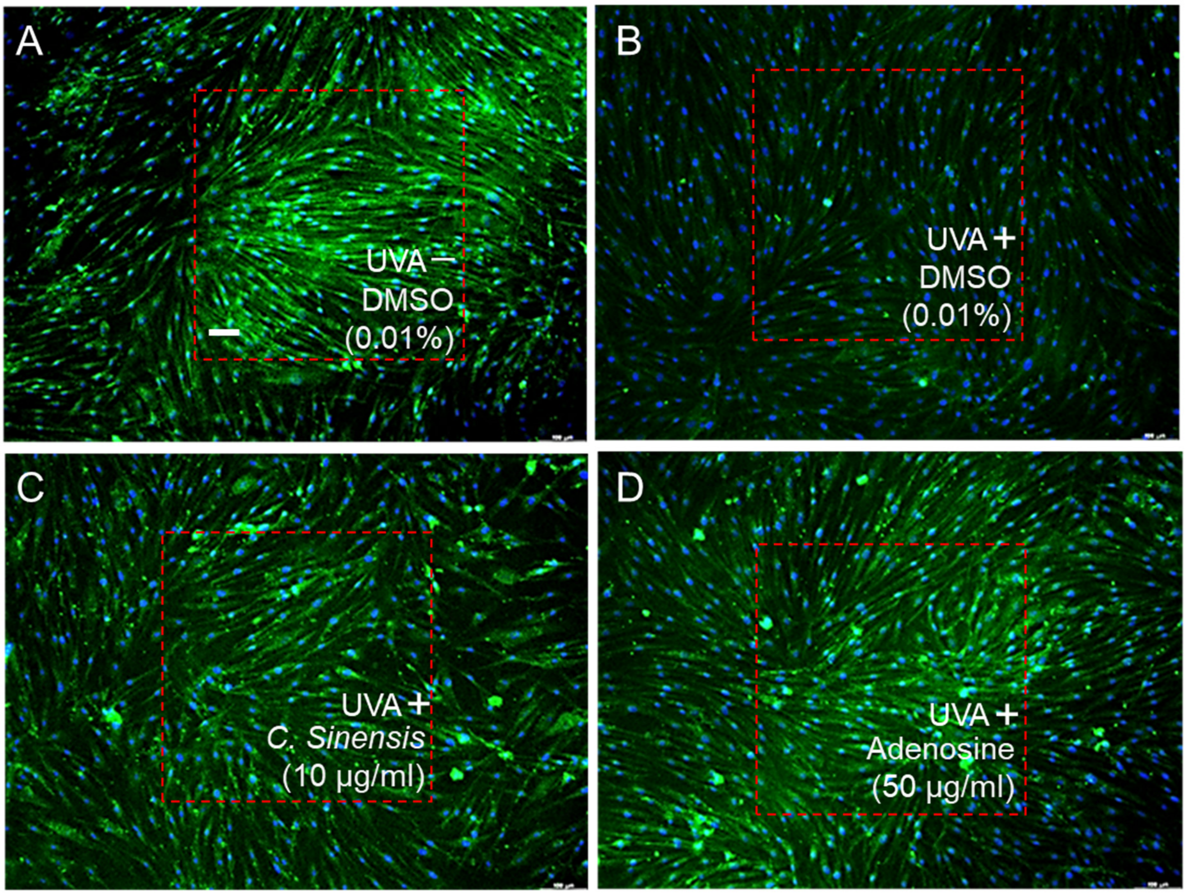

Supplementary Figure S1. Full-size image of immunofluorescence in Figure 5C. (A) Original image of DMSO (UVA−) in Figure 5C. (B) Original image of DMSO (UVA+) in Figure 5C. (C) Original image of C. sinensis (UVA+) in Figure 5C. (D) Original image of Adenosine (UVA+) in Figure 5C.

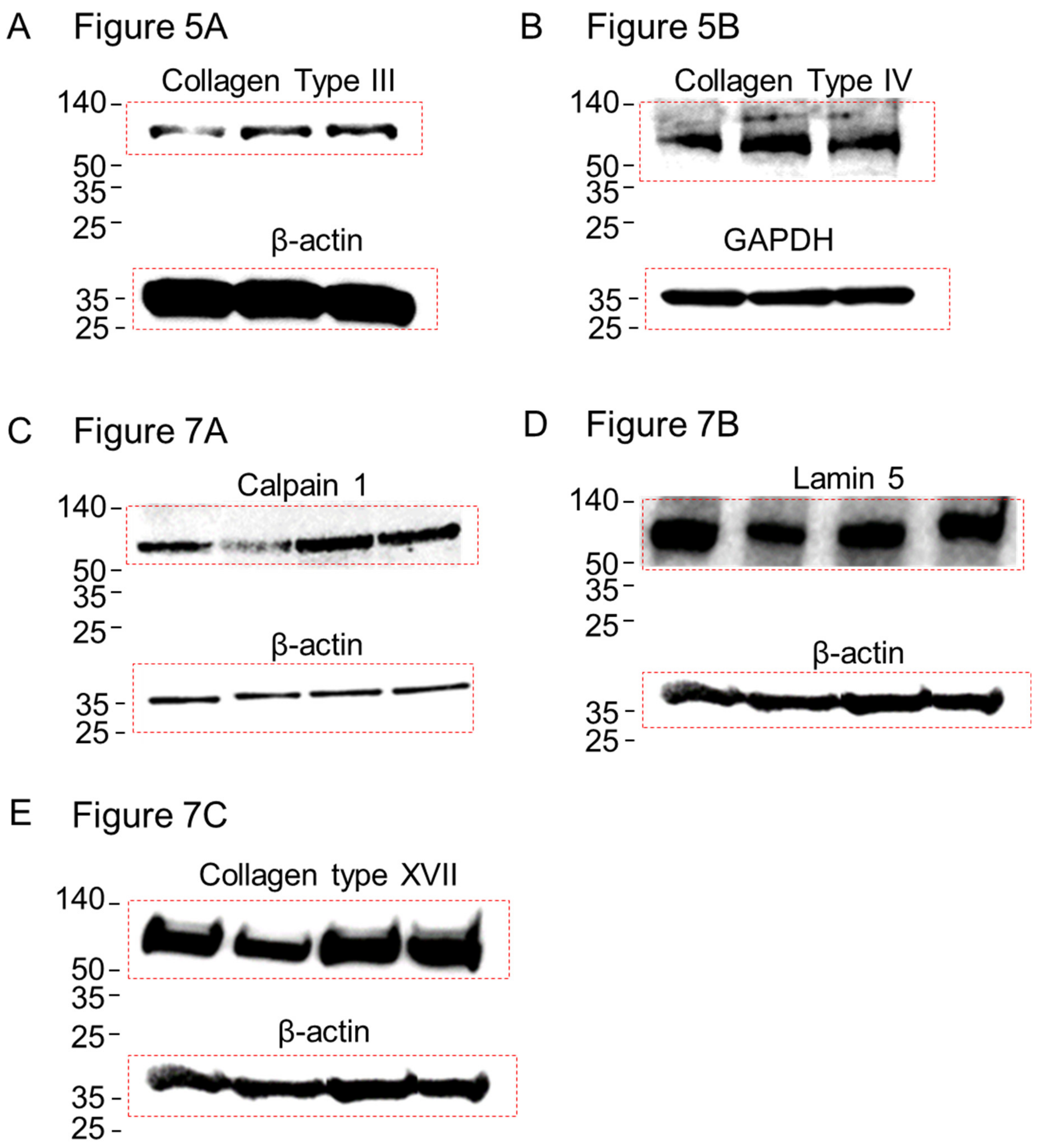

Supplementary Figure S2. Full-size image of western blot. (A) Full size image in Figure 5A. (B) Full size image in Figure 5B. (C) Full size image in Figure 7A. (D) Full size image in Figure 7B. (E) Full size image in Figure 7C.
